# The Impact of Reintervention on Arteriovenous Fistula Maturation and Functional Patency in the Hemodialysis Fistula Maturation Study

**DOI:** 10.1016/j.xkme.2025.101036

**Published:** 2025-05-26

**Authors:** Erik M. Anderson, Thomas S. Huber, Dan Neal, Scott A. Berceli, Samir K. Shah, David H. Stone, Salvatore T. Scali, Hemodialysis Fistula Maturation Study Group

**Affiliations:** 1 Division of Vascular Surgery and Endovascular Therapy, University of Florida, Gainesville, FL; 2 Section of Vascular Surgery, Dartmouth-Hitchcock Medical Center, Lebanon, NH

**Keywords:** Arteriovenous fistula, dialysis access, fistula maturation, reintervention, vascular access

## Abstract

**Rationale & Objective:**

Arteriovenous dialysis fistula (AVF) reinterventions are sought to assist with maturation and/or maintain functional patency; however, their ultimate impact on fistula lifespan remains poorly documented. Furthermore, current clinical practice guidelines lack clarity regarding reintervention paradigms to achieve optimal AVF performance. Therefore, the purpose of this study was to document reintervention rates and their association with AVF maturation and functional patency among patients enrolled in the Hemodialysis Fistula Maturation (HFM) study.

**Study Design:**

Retrospective review of a prospective observational cohort study.

**Setting & Participants:**

In total, 535 HFM study patients who underwent maturation adjudication across 7 academic centers.

**Exposures:**

Pre- and postmaturation reinterventions (ie, revisional procedures) for single-stage, upper extremity AVFs.

**Outcomes:**

AVF reintervention-associated maturation success and functional patency.

**Analytical Approach:**

Descriptive statistical methods, including Kaplan–Meier methodology, characterized unadjusted reintervention outcomes.

**Results:**

In total, 396 (74%) AVFs were successfully used for dialysis, and 37% (N=196 out of 535) underwent 274 reinterventions (181 endovascular, 93 open) to facilitate maturation. Factors associated with prematuration reintervention included female sex, diabetes, peripheral vascular disease, and elevated body mass index. Following maturation, 47% (N=188 out of 396) of the patients with a functional AVF underwent 477 reinterventions. The postmaturation reintervention clinical success rate was 70% (endovascular 72% [N=312 out of 435]; open 55% [N=23 out of 42]). Assisted maturation AVFs demonstrated inferior functional primary patency (*P* = 0.002) but equivalent cumulative functional patency (*P* > 0.9) compared with unassisted maturation fistulas. Postmaturation abandonment rate was 24% (N=95 out of 395).

**Limitations:**

AVF management decisions were made by the individual surgeons, so this study cannot account for physician and center selection bias related to access use, remediation, or abandonment. Furthermore, AVFs were exclusively managed at academic institutions, so results may not be generalizable across all health care settings. Finally, prosthetic conduits were not evaluated.

**Conclusions:**

AVF reinterventions are common and are not associated with inferior maturation or functional patency rates. Timely remediation should be considered when clinically indicated, although AVFs remain at high-risk for subsequent reinterventions, with durable outcomes requiring meticulous surveillance.

## Plain Language Summary

The Hemodialysis Fistula Maturation (HFM) study was reviewed to analyze how often arteriovenous fistulas (AVFs) required additional procedures (reinterventions) and how successful these procedures were in ensuring functional access. Reinterventions were categorized as either prematuration (to promote AVF maturation) or postmaturation (to address AVF malfunction). More than one-third of AVFs required a prematuration reintervention, with about half of these being successful. Patients at higher risk for requiring interventions included those who were female, had diabetes, peripheral vascular disease, or a high body mass index. Postmaturation reinterventions were performed in about half of mature AVFs, with a success rate of 70%. Although AVFs needing prematuration interventions had lower rates of freedom from reintervention after maturation, their overall functional patency was comparable with AVFs that did not require early intervention. These findings suggest that AVF reinterventions are common but do not compromise long-term access durability.

Historically, widespread underutilization of arteriovenous fistula (AVF) creation for patients with hemodialysis dependent kidney disease prompted national society guidelines to incentivize this type of autogenous access.^1^ The rationale for such efforts was predicated on their superior patency, decreased infection, and improved survival rates among AVF recipients compared with patients receiving alternative access types.^2^^,^^3^ Unfortunately, the increased emphasis on AVF creation led to the unintended consequence of correspondingly high nonmaturation rates (∼30% to 60%).^4^, ^5^, ^6^, ^7^, ^8^ Not surprisingly, multiple studies have reported high reintervention rates to facilitate both maturation and AVF functional patency (ie, percutaneous or surgical procedures, after initial AVF creation, performed before or after successful cannulation for dialysis).^9^, ^10^, ^11^, ^12^ Interestingly, these reports have raised concerns that the perceived superiority of autogenous access may be somewhat compromised because autogenous reintervention rates have approached those of AV grafts.^13^

Accordingly, there is a lack of consensus as to the optimal access treatment paradigm. Specifically, rather than a “one size fits all” strategy for AVF utilization, recently updated Kidney Disease Outcomes Quality Initiative (KDOQI) guidelines now advocate for the “right access, in the right patient, at the right time, for the right reasons.”^14^ Despite this paradigm change, AVFs remain the most prevalent surgical access subtype.^15^ Moreover, clinical practice guidelines lack granular information surrounding reinterventions and their anticipated impact on outcomes to guide postoperative decision making. Importantly, the effects of reintervention on AVF lifespan remain poorly described. Pre- and postmaturation reinterventions are often not differentiated in the literature, and reported technical success rates of 80%-98% unfortunately do not reflect function outcomes (eg, successful AVF maturation, successful resumed or continued dialysis use).^16^, ^17^, ^18^ Additionally, recent data from the US Renal Data System conflicts with prior reports that AVFs requiring maturation assistance have inferior cumulative functional patency rates.^19^, ^20^, ^21^

Notably, because of increased documented AVF nonmaturation rates, the Hemodialysis Fistula Maturation (HFM) study was conducted to elucidate the biological, surgical, and system-based factors that foster fistula maturation.^22^^,^^23^ This landmark study generated the most rigorously adjudicated and robust longitudinal observational dataset to date and provides contemporary AVF maturation, reintervention, and functional outcome data. Huber et al^23^ reported a 1-year AVF maturation rate of 76% for 535 AVFs across 7 centers, with a functional patency rate of 75% at 2 years. In total, 37.7% of kidney failure patients and 34.6% of chronic kidney disease patients required maturation assistance, and 47.5% of all patients underwent postmaturation reinterventions.

Therefore, the purpose of this study was to document results from the HFM study to better characterize the type, frequency, and midterm outcomes of AVF reinterventions and their association with maturation and functional patency. Furthermore, we sought to describe clinical success rates of different reinterventions to better inform management of failing AVFs.

## Methods

This study was approved by the University of Florida Institutional Review Board (#1722-2022), and a waiver of informed consent was granted because no patient contact or study-related interventions occurred. The Strengthening the Reporting of Observational Studies in Epidemiology guidelines for observational research were followed.^24^

### HFM Study

The HFM study was a multicenter (N=7), prospective observational cohort study (https://repository.niddk.nih.gov/studies/hfmc/).^23^ The primary objective of the study was to improve understanding of AVF maturation failure by examining the relationship among clinical usability, anatomy, biology, clinical attributes, and processes of care.^22^ Adults with planned single-stage upper extremity fistulas were included, and clinical decisions regarding AVF creation and postoperative management were deferred to care teams at each institution. Reinterventions within the first 6 weeks of AVF creation were discouraged per the study protocol. The primary endpoint of the HFM study was AVF maturation, which was defined as effective 2-needle cannulation use for ≥75% of dialysis sessions over a 4-week period. A dedicated committee reviewed study records to adjudicate maturation outcomes (success or failure) and assign a maturation date, when appropriate.

### Study Population

Both active (kidney failure) and anticipated (chronic kidney disease; CKD stage 5) adult (age >18 years) hemodialysis patients were recruited to participate in the study. Additionally, an expectation of dialysis initiation within 3 months of AVF placement for patients with CKD was necessary for enrollment. AVF maturation was not assessed in CKD participants until they initiated dialysis. Demographics and comorbid conditions were established at time of registration using predefined criteria.^22^ Patients were monitored monthly during study participation or for at least 3 months after fistula abandonment.

### Endpoints and Definitions

AVF creation was considered the incident intervention for each patient, so any subsequent remedial procedure was considered a “reintervention.” Reinterventions included surgical or percutaneous procedures to treat AVF malfunction, such as fistula stenosis, inflow or outflow stenosis, thrombosis, accessory vein branches, and inability to cannulate or to treat complications, including hand ischemia, hematoma/seroma, thrombosis, etc. Diagnostic studies without additional procedures, such as fistulagrams, were not included as a reintervention. The primary endpoint for this analysis was the incidence of AVF reintervention before and after maturation. A secondary endpoint was occurrence of AVF abandonment. Prematuration reinterventions were considered clinically successful if the fistula matured without subsequent reinterventions. Postmaturation reinterventions were considered successful if effective hemodialysis was continued or resumed within 3 months of the remedial procedure.

Reinterventions with multiple concurrent procedures were assigned a primary indication/procedure type to avoid duplicating success and failure judgments for a single reintervention. Assignment of a primary indication was based on a predetermined hierarchy. “Thrombosis” was ascribed the highest priority indication, followed by indications to facilitate maturation (“fistula stenosis,” “central venous stenosis,” “inflow stenosis,” “accessory vein branch,” and “inability to cannulate”), and lastly, indications to manage complications (“hand ischemia,” “bleeding,” “fluid evacuation,” “infection,” and “aneurysm”). For example, if a patient underwent accessory vein branch coil embolization and fistula balloon angioplasty during one reintervention, fistula stenosis would be considered the primary indication. All primary indication/procedure assignments were reviewed by the study team for accuracy.

As previously described, the prematuration success or failure endpoint was adjudicated by the HFM study steering committee.^10^^,^^22^^,^^23^ Successful maturation was further categorized as assisted (eg, reintervention prematuration) or unassisted (eg, no reintervention prematuration). After an AVF achieved maturation and clinical usability, postmaturation endpoints included functional patency or abandonment, which were determined by fistula use at last known follow-up. “Functional primary patency” was defined as a functional AVF that did not undergo any reintervention to maintain and/or re-establish patency. “Cumulative functional patency” was defined by the total time of clinical usability for an AVF before abandonment (or study completion). Given potential ambiguity, traditional terms such as “primary patency” and “secondary(cumulative) patency”, were not used, and terminology for this study matches definitions from current KDOQI guidelines.^14^

### Statistical Analysis

Participant and procedure characteristics are presented as N(%) for categorical variables and mean (± standard deviation [SD]) or median (25th, 75th percentiles) for continuous variables. Fisher exact tests and *t* tests were used to make group comparisons on categorical and continuous variables, respectively. Kaplan–Meier methods were used to characterize time to first reintervention, time to maturation, and time to loss of patency, and log-rank tests were used to compare groups on these outcomes. Because HFM data consist of a nonrandom sample from a limited number of treatment facilities, its suitability for generalization to the population of all hemodialysis patients is uncertain. Therefore, sample means and proportions are reported without confidence intervals or risk adjustment and are not intended as population estimates. *P* values are reported only to facilitate interpretation of group differences observed among HFM study participants, not to establish statistically significant differences in a larger population. Linear models (linear, logistic, and Cox regression, as appropriate) controlling for age, sex, race, body mass index (BMI), smoking status, diabetes, coronary artery disease, peripheral artery disease, and dyslipidemia were used to risk-adjust these comparisons. All analyses were performed on the original study data using the R statistical software package (V.4.0.2, the R Foundation for Statistical Computing).

## Results

### Patient Cohort

Maturation adjudication was ascertained in 535 patients with AVF in the HFM study. The mean age was 55 (±14) years, and a majority were male (70%). Pre-emptive access placement occurred in 34%. Overall, a majority had diabetes (58%), and the average BMI was 30 (±8). The most common fistula location was the upper arm (64%), and the study access was the first surgical access in 78% of patients. Notably, 63% had a tunneled dialysis catheter (TDC) at time of AVF placement.

### Maturation and Functional Patency

**Figure 1:**
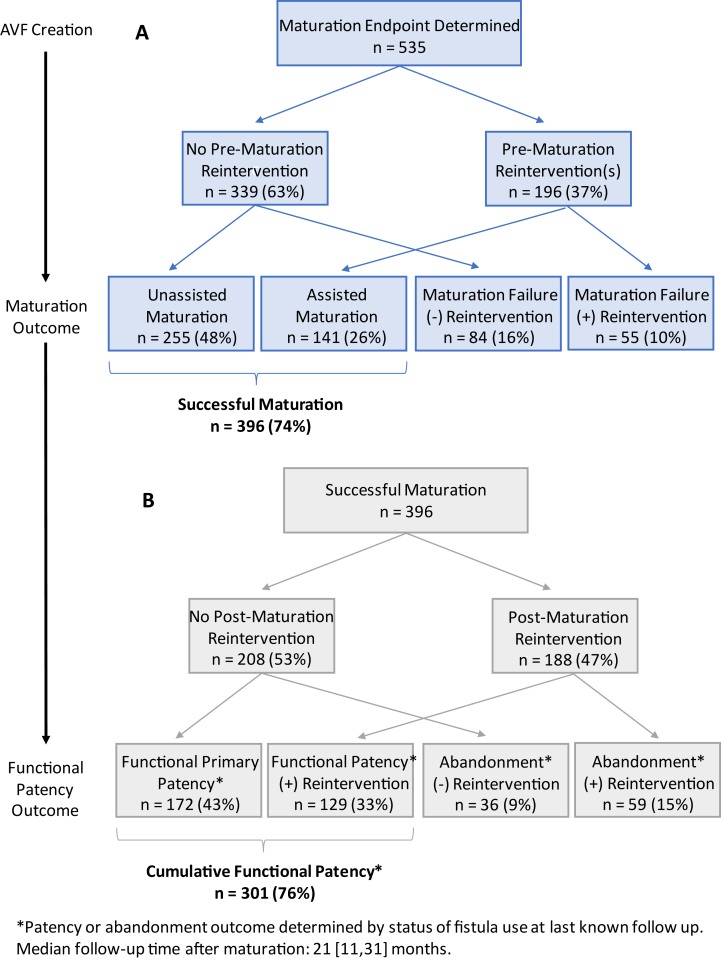
Study cohort before (A) and after (B) maturation outcome. The Hemodialysis Fistula Maturation (HFM) study cohort is depicted in the diagram. Panel (A) highlights the maturation outcomes for arteriovenous fistulas (AVFs) in the HFM study stratified by whether or not a reintervention occurred before clinical maturation. Note, timeline to maturation was individualized and based successful clinical use as adjudicated by the HFM steering review committee. In total, 74% of all AVFs ultimately achieved successful cannulation (eg, 2 needle cannulation for at least 75% of dialysis sessions over a 4 week period). Panel (B) features the ultimate clinical outcomes (eg, functional patency or abandonment) for the subset of AVFs (N=396) that achieved maturation and then either did or did not undergo postmaturation reintervention. Patency or abandonment outcome was determined by the status of the AVF use at last known follow-up. Median follow-up time after maturation was 21 [IQR, 11-31] months.

Figure 1A depicts the maturation and functional patency outcomes for all 535 adjudicated patients. The overall study successful maturation rate was 74% (N=396). Approximately half (48%, N=255) experienced unassisted maturation, whereas 16% (N=84) failed without undergoing reintervention. One or more prematuration reinterventions were performed in 37% (N=196 out of 535). Among the 396 AVFs that achieved successful maturation, 141 (36%; 26% overall) were assisted. The median time from access placement to maturation was greater for participants undergoing reintervention (5 [interquartile range {IQR}: 4-8] vs unassisted maturation, 3 [IQR, 3-4] months; *P* < 0.0001). The risk-adjusted estimate was 39% higher for reintervention patients (95% confidence interval [CI]: 24.5% to 56.2%; *P* < 0.001). Similarly, the median TDC time was longer for assisted maturation fistulas (3 [IQR, 2-5] vs unassisted maturation, 2 [0-3] months; *P* < 0.0001). The risk-adjusted estimate was 96% higher for reintervention patients (95% CI, 44.4%-166.0%; *P* < 0.001).

Fig 1B highlights the functional patency rates for patients attaining successful maturation (N=396). The median follow-up time postmaturation was 21 (IQR, 11-31) months. In total, 43% (N=172 out of 396) experienced continuous AVF usability without postmaturation reintervention (eg, “functional primary patency”). The remaining patients either underwent postmaturation reintervention (48%, N=188 out of 396) or abandonment without reintervention (9%, N=36). Among patients receiving postmaturation reintervention, 33% (N=129) had continued AVF use during follow-up; however, 15% (N=59) were abandoned after attempted revision. In total, 24% (N=95) of mature AVFs were ultimately abandoned at a median time of 16 (IQR, 8-26) months after maturation. There was no difference in cumulative functional patency between CKD and kidney failure patients (CKD: 76%, N=97 out of 127 vs kidney failure: 76%, N=204 out of 269; risk-adjusted estimate: HR for fistula abandonment = 1.0 [95% CI, 0.64-1.65; *P* = 0.90]). Table S1 includes distribution of patient characteristics, maturation outcomes, and functional patency outcomes by center.

### Prematuration Reintervention

Characteristics of patients exposed to prematuration reintervention (N=196) compared with those without reintervention (N=339) are depicted in Table 1. Notably, female sex, higher BMI, diabetes, and peripheral artery disease were all more prevalent among reintervention patients. However, successful maturation rates were similar between prematuration intervention groups (reintervention 72%, no reintervention 75%, risk-adjusted estimate: odds ratio [OR] = 0.99 [95% CI, 0.65-1.53; *P* = 0.90]). Furthermore, maturation success rates were not influenced by the number of reinterventions(*P* = 0.50) (Table S2). Among AVFs failing to mature postreintervention, female sex was the only significant covariate (*P* = 0.005) (Table 2). Although dialysis status (eg, CKD vs kidney failure) was not associated with prematuration reintervention frequency or maturation success rate after reintervention, median time to first reintervention was longer for patients with CKD (4 [IQR, 2-5]; kidney failure 3 [IQR, 2-4] months). Similarly, as expected, patients with CKD had longer median time to maturation (7 [IQR, 5-9]; kidney failure 5 [IQR, 4-6] months).

**Table 1:** Demographics and Comorbid Conditions Stratified by Prematuration Intervention Status.

Variable, No. (%)	No Reintervention N=339 (63%)	Reintervention N=196 (37%)	*P* Value
Age, y (mean±SD)	54 ± 14	56 ± 13	0.1
Female sex	91 (27%)	72 (37%)	0.02
Race			0.5
White	148 (44%)	96 (50%)	
Non-White	186 (56%)	97 (50%)	
BMI (mean±SD)	29.4 ± 7.4	31.8 ± 7.6	0.0005
Smoking status			0.2
Never	161 (48%)	89 (45%)	
Former	111 (33%)	79 (40%)	
Current	62 (19%)	28 (14%)	
Diabetes mellitus	186 (55%)	125 (64%)	0.05
Coronary artery disease	77 (23%)	58 (30%)	0.08
Peripheral artery disease	41 (12%)	41 (21%)	0.009
Dyslipidemia	189 (56%)	110 (56%)	1.0
CKD/Pre-emptive access placement	119 (35%)	63 (32%)	0.5
Prior vascular access	78 (23%)	40 (21%)	0.5
Forearm arteriovenous fistula	96 (28%)	47 (24%)	0.3

Abbreviations: BMI, body mass index; CKD, chronic kidney disease (stage V); SD, standard deviation.

**Table 2:** Demographics and Comorbid conditions of Prematuration Fistulas Undergoing Reintervention (N=196) Stratified by Ultimate Maturation Outcome.

Variable, No. (%)	Successful Maturation N=141 (72%)	Failed Maturation N=55 (28%)	*P* Value
Age, y (mean±SD)	55 ± 13	57 ± 14	0.5
Female sex	43 (31%)	29 (53%)	0.005
Race			0.9
White	70 (50%)	26 (48%)	
Non-White	69 (50%)	28 (52%)	
BMI (mean±SD)	31 ± 7	33 ± 9	0.1
Smoking status			0.8
Never	64 (45%)	25 (46%)	
Former	58 (41%)	21 (38%)	
Current	19 (14%)	9 (16%)	
Diabetes mellitus	86 (61%)	39 (71%)	0.2
Coronary artery disease	39 (28%)	19 (35%)	0.4
Peripheral vascular disease	28 (20%)	13 (24%)	0.6
Dyslipidemia	79 (56%)	31 (56%)	1.0
CKD/Pre-emptive access placement	44 (31%)	19 (35%)	0.7
Prior vascular access	33 (24%)	7 (13%)	0.1
Forearm arteriovenous fistula	37 (26%)	10 (18%)	0.3

Abbreviations: BMI, body mass index; CKD, chronic kidney disease (stage V); SD, standard deviation.

### Prematuration Reintervention Outcomes

Among the 196 patients undergoing prematuration remediation, 274 reinterventions (endovascular 66% [N=181]; open 34% [N=93]) were performed with an average of 1.4 ± 0.7 reinterventions per subject (0.14 interventions per preadjudication month per person) and 1.3 ± 0.5 procedures per reintervention. The median time to first reintervention was 3 [IQR, 2-4] months for both open and endovascular procedures. The overall success rate for prematuration reinterventions was 51%, with similar outcomes for open (53%, N=49 out of 93) and endovascular (51%, N=92 out of 181) procedures.

**Figure 2:**
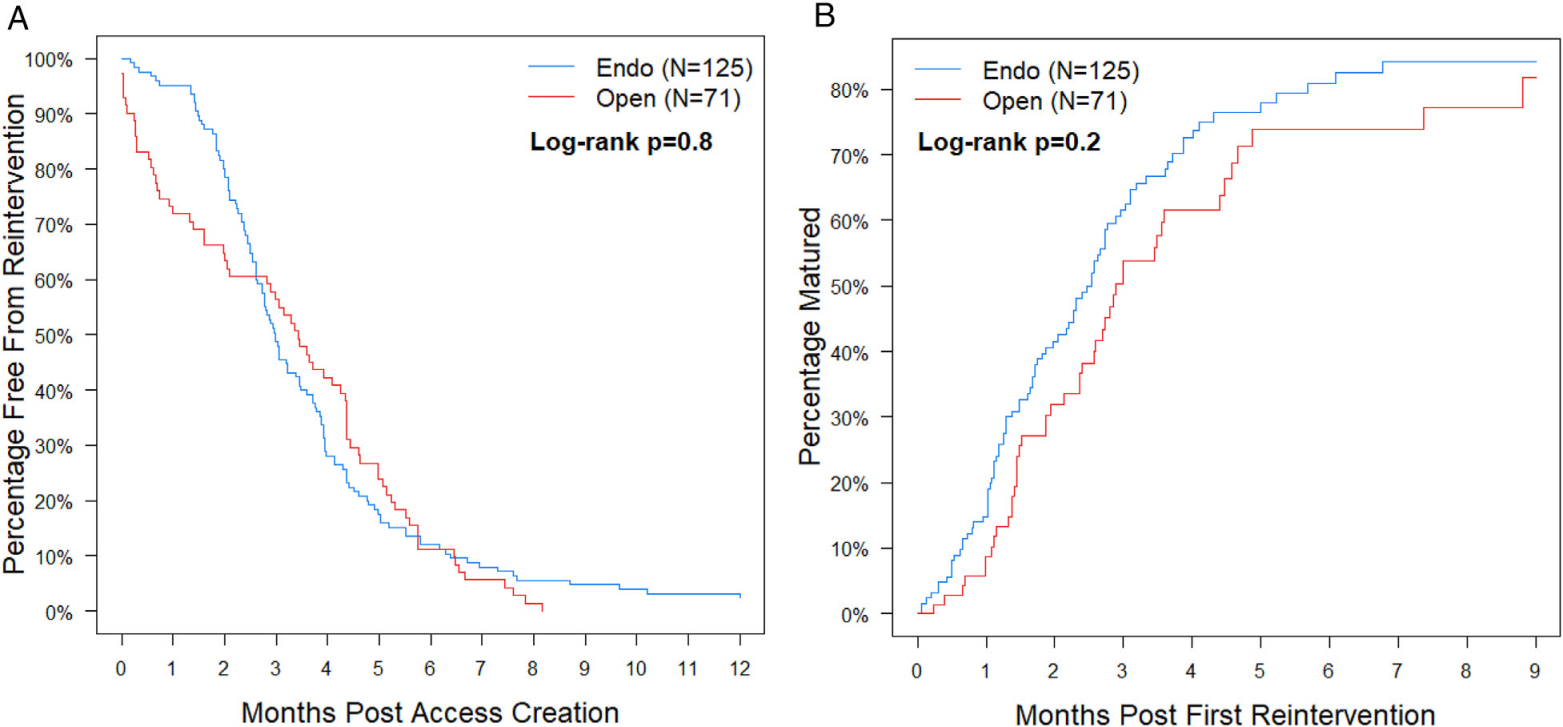
Cumulative incidence curves for time from access creation to first reintervention (A) and time from first intervention to maturation (B). Focusing on the arteriovenous fistulas in the Hemodialysis Fistula Maturation Study that underwent reintervention prematuration (n=196), the timing of first reintervention did not differ by procedure subtype (open versus endovascular, *P* = 0.80). Similarly, no difference between procedure subtypes and the time from first reintervention to eventual maturation was observed (*P* = 0.20).

Table 3 lists all prematuration reinterventions by primary indication and procedure type, as well as success rates. Most reinterventions were endovascular procedures performed for fistula stenosis (N=149 out of 274). Both endovascular and open procedures had comparable success in treating fistula stenosis (53%), thrombosis (38%), and central vein stenosis (50%). Types of reintervention and success rates did not differ between CKD and patients with kidney failure (Table S3). Compared with upper arm AVFs, forearm fistulas more commonly received reinterventions for accessory vein branches or inability to cannulate but conversely did not undergo any inflow stenosis reinterventions (Table S4). Fig 2A depicts the freedom from reintervention for AVFs (N=196) that underwent either endovascular or open remediation as their index procedure. Temporal differences in reintervention type were evident because open procedures predominated during the first 2 postoperative months, whereas endovascular procedure frequency increased after 6 weeks. Time from first reintervention to maturation did not differ significantly by procedure type (risk-adjusted estimate: hazard ratio [HR] for maturation open vs endo = 0.79 [95% CI, 0.53-1.17; *P* = 0.20]) (Fig 2B). Table S5 depicts center-specific prematuration reinterventions and success rates.

**Table 3:** Indications and Types of Reinterventions Performed on Arteriovenous Fistulas Pre- and Postmaturation.

Primary Indication/Procedure Type	Prematuration Interventions (N=274 Interventions in 196 Patients)		Postmaturation Interventions (N=477 Interventions in 188 Patients)	
	N Interventions (% of total)	Proportion Successful (%)	N Interventions (% of total)	Proportion Successful (%)
Fistula Stenosis	166 (61%)	88 (53%)	326 (68%)	251 (77%)
Endovascular (eg, angioplasty, stent)	149	79	318	244
Open (eg, patch angioplasty, bypass)	17	9	8	7
Thrombosis	21 (8%)	8 (38%)	63 (13%)	23 (37%)
Endovascular (eg, percutaneous thrombectomy)	13	5	52	20
Open (eg, open thrombectomy)	8	3	11	3
Accessory vein branches	19 (7%)	9 (47%)	7 (1%)	7 (100%)
Endovascular (eg, embolization)	1	0	6	6
Open (eg, ligation)	18	9	1	1
Central vein stenosis	18 (7%)	9 (50%)	49 (10%)	35 (71%)
Endovascular (eg, angioplasty, stent)	14	7	49	35
Open (eg, patch angioplasty, bypass)	4	2	0	0
Inability to cannulate	15 (5%)	12 (80%)	2 (<1%)	1 (50%)
Endovascular	0	0	0	0
Open (eg, superficialization)	15	12	2	1
Inflow stenosis	13 (4%)	6 (46%)	8 (2%)	7 (88%)
Endovascular (eg, angioplasty, stent)	3	1	7	6
Open (eg, patch angioplasty, bypass)	10	5	1	1
Hand ischemia	16 (6%)	5 (31%)	5 (1%)	3 (60%)
Endovascular	0	0	0	0
Open (eg, ligation, revision)	16	5	5	3
Other (bleeding, fluid evacuation, infection, aneurysm)	6 (2%)	4 (67%)	17 (4%)	8 (47%)
Endovascular	1	0	3	1
Open	5	4	14	7
Total	274 (100%)	141 (51%)	477 (100%)	335 (70%)
Endovascular	181	92	435	312
Open	93	49	42	23

### Postmaturation Reintervention

**Figure 3:**
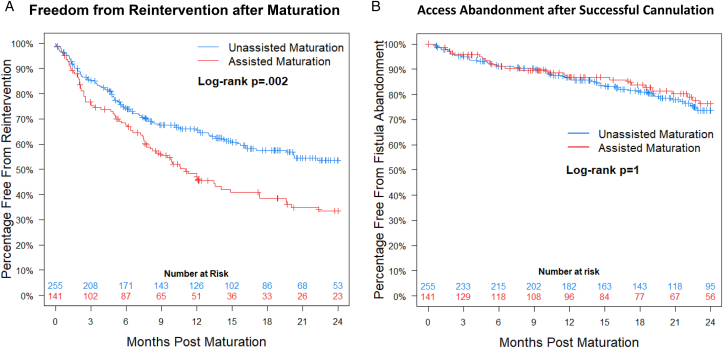
Kaplan–Meier curves depicting freedom from reintervention after maturation (A) and timing of access abandonment after successful cannulation (B). Focusing on arteriovenous fistulas in the Hemodialysis Fistula Maturation Study that successfully matured (n=396), functional primary patency was worse for fistulas that underwent reintervention before maturation (*P* = 0.002). Despite this increased frequency of postmaturation interventions, fistulas undergoing reintervention before maturation did not have an increased rate of abandonment (*P* = 1.0). Figure 3B republished from *JAMA Surgery* with permission.^20^

Assisted maturation AVFs underwent more reinterventions after initial successful cannulation for dialysis (risk-adjusted estimate: HR = 1.5 [95% CI, 1.14-2.11; *P* = 0.005]) (Fig 3A).^20^ One-year freedom from reintervention (eg, 1-year functional primary patency) was 47 ± 5% for assisted maturation vs 66 ± 3% for unassisted maturation. However, median time to first postmaturation reintervention was the same between groups (5 [IQR, 2-10] months). Further, assisted maturation fistulas had equivalent cumulative functional patency (risk-adjusted estimate: HR for loss of patency = 0.92 [95% CI, 0.58-1.45; *P* = 0.70]) (Fig 3B).^20^ At last known follow-up, freedom from abandonment was 77% (N=108 out of 141) for assisted maturation AVFs and 76% (N=193 out of 255) for unassisted maturation AVFs.

Interestingly, cumulative functional patency was influenced by the number of postmaturation reinterventions (Table S6). Specifically, patients who did not undergo any postmaturation reinterventions had a functional patency rate of 83%. Nearly one-third of patients (29%, N=113) underwent multiple postmaturation reinterventions. Patients with ≥3 postmaturation reinterventions (18%, N=71) had comparable functional patency rates, ranging from 73% to 93%. In fact, AVFs with ≥6 reinterventions had the greatest long-term functional patency (93%). Alternatively, patients who received 1-2 postmaturation reinterventions had inferior functional patency rates (60%-62%) (risk-adjusted estimate: HR for abandonment 1 or 2 vs none = 1.9 [95% CI, 1.16-3.01; *P* = 0.011], HR ≥ 3 vs 1 or 2 = 0.29 [95% CI, 0.14-0.57; *P* < 0.001]). High reintervention patients (5 or more pre- or postmaturation reinterventions; n=44) more often had peripheral artery disease (*P* = 0.03) and had upper arm AVF creation (vs forearm, *P* = 0.002) (Table S7).

### Postmaturation Reintervention Outcomes

A total of 477 postmaturation reinterventions were performed (mean 2.5 ± 1.9 reinterventions per patient and 1.4 ± 0.6 procedures per reintervention event) (Table 3). A majority of postmaturation reinterventions were endovascular (91%, N=435 out of 477), which is a higher proportion than prematuration reinterventions (endovascular, 66%, N=181/274). Analogous to prematuration reinterventions, a majority of the remedial procedure indications were for fistula stenosis (68%), followed by interventions to address thrombosis (13%) and central vein stenosis (10%), with slightly increased relative frequency compared with prematuration reinterventions. Similarly, procedures managing complications (eg, bleeding, fluid evacuation, infection, aneurysm) increased in frequency postmaturation (4% vs 2%).

The overall postmaturation reintervention clinical success rate was 70% (N=335 out of 477), which is higher than the prematuration reintervention rate of 51% (Table 3). This was because of efficacious endovascular reinterventions (72% [N=312 out of 435] vs prematuration, 51% [N=92 out of 181]). Conversely, open procedures had comparable success rates (prematuration, 53% [N=49 out of 93]; postmaturation, 55% [N=23 out of 74]). Endovascular procedures to address AVF stenosis constituted a majority of the postmaturation reintervention successful outcomes (77%, N=244 out of 318). Table S8 depicts center-specific postmaturation reinterventions and success rates.

## Discussion

This study is the first descriptive analysis to characterize clinical functional success rates of pre- and postmaturation AVF reinterventions using the HFM study population. These results can be used to better inform clinical decision making to address problems impairing AVF maturation or complications after successful cannulation. It should be noted that a majority of patients achieved successful maturation (74%) and that reinterventions were often necessitated to promote maturation (37%) or maintain functional patency (48%). The proportion of AVFs in this analysis with assisted maturation is similar to previous reports (∼31% to 51%).^9^, ^10^, ^11^^,^^16^^,^^19^, ^20^, ^21^ However, functional primary patency was higher among the HFM cohort compared with other studies (∼22% to 44%).^12^^,^^21^ Ultimately, we determined that pre- and postmaturation reinterventions did not appear to reduce cumulative functional patency in this study.

Variables associated with prematuration reintervention included female sex, diabetes, peripheral vascular disease, and elevated BMI. Among these factors, only female sex was associated with increased maturation failure rates after reintervention. The higher incidence of reinterventions and reintervention failures should be considered for female patients when planning autogenous access creation or considering remediation for a failing access. Miller et al^11^ has previously highlighted sex-based differences related to AVF maturation and reinterventions. Interestingly, dialysis status at time of AVF placement and access location did not influence prematuration reintervention rates, similar to other reports.^11^^,^^20^^,^^21^ Additionally, a prior failed vascular access did not increase risk of prematuration, reintervention, or probability of maturation failure after reintervention. This finding is similar to Lee et al,^20^ who demonstrated that a first time AVF did not have fewer prematuration reinterventions than a subsequent access, suggesting that subsequent AVFs in the same patient will likely have independent predictors of nonmaturation.

To guide management of AVF nonmaturation, we sought to evaluate prematuration reintervention success rates by procedure indication and type. More than half of the reinterventions in the HFM study population were performed for fistula stenosis (61%), with thrombosis, accessory vein branch, and central vein stenosis the next most common indications. This is analogous to Falk et al^16^ who reported that 59% of prematuration procedures were performed for fistula stenosis. Although, in a smaller study by Miller et al,^11^ accessory vein ligation predominated. Notably, Miller et al^11^ documented that accessory vein ligation was used more frequently for forearm AVFs, which comprised almost half of their access experience. We similarly found a higher proportion of accessory vein ligation procedures for forearm AVFs, although the procedures were less often successful in the forearm (50% vs 67%), likely because of the more dense venous collateralization network in the forearm. Forearm AVFs also had a higher proportion of thrombectomies and procedures for inability to cannulate than upper arm AVFs. High reintervention patients (5+ reinterventions) more commonly had upper arm AVFs, which suggests that failing forearm AVFs do not undergo aggressive attempts at sequential remediation because of the alternative option of a more proximal AVF.

Open surgical procedures were most common during the first 2 months after AVF placement; however, endovascular procedures increased in frequency after 6 weeks. As expected, this suggests that open reinterventions are often used to manage postoperative complications, whereas endovascular reinterventions are preferentially used after 6 weeks to address nonmaturing fistulas, typically because of neointimal hyperplastic disease. Although most remediations were endoluminal-based therapies to promote maturation (66%), procedure type did not influence time to maturation or reintervention success rates (51% endovascular; 53% open). Similar to our results, a review of 173 AVFs found a preponderance of endovascular reinterventions (71%) before maturation but no outcome disparities among procedure types.^20^ These findings suggest that the optimal approach to management of a failing access should be guided by clinical and anatomical characteristics contributing to the underlying etiology for nonmaturation.

Although a 51% prematuration reintervention success rate is sobering, the proportion of patients achieving successful maturation after one or more reinterventions was good (72%) and underscores a proactive approach to AVF maintenance. Some patients received multiple reinterventions before maturation adjudication, which accounts for this discrepancy (eg, if successful maturation occurred, only the last reintervention was considered “successful”). Interestingly, the number of reinterventions did not influence the maturation rate. Patients without any prematuration reinterventions did not have superior maturation rates compared with patients with ≥1 reinterventions (75% vs 71%). Further, an increasing number of reinterventions did not appear to negatively affect maturation rates.

In contrast, multiple reinterventions were common in the postmaturation phase and affected cumulative functional patency rates. Indeed, approximately one-third (29%) of patients underwent ≥2 postmaturation reinterventions. These findings are similar to Bountouris et al,^25^ in which ∼50% of AVFs receiving percutaneous reintervention had multiple procedures. Remarkably, AVFs with 0 or ≥3 reinterventions had superior functional patency compared with those with 1 or 2 reinterventions in our study. In fact, the highest functional patency rate occurred among AVF with ≥6 reinterventions (93%), which was superior to fistulas without any reinterventions (83%). Likely, this bimodal distribution suggests 2 distinct populations once a fistula successfully matures: patients who have no issues with their fistula and patients who require sequential reinterventions to maintain functional patency. Malka et al^17^ similarly reported that AVFs with multiple percutaneous reinterventions had excellent 5-year functional patency (>85%).

In this analysis, assisted maturation AVFs did not experience inferior cumulative functional patency compared with unassisted maturation AVFs. Although 2 smaller studies reported contrary results, Lee et al.’s^20^^,^^21^ examination of the US Renal Data System found that prematuration reinterventions did not compromise total access usability time, similar to our findings.^19^ Despite an equivalent AVF lifespan, assisted maturation fistulas demonstrated longer time to maturation adjudication. Not surprisingly, this correlated with increased TDC time. Additionally, previous studies collectively agree with our findings that assisted maturation AVFs are associated with increased frequency of postmaturation reinterventions.^19^, ^20^, ^21^

Taken together, these results support the concept that the effect of reintervention before and/or after AVF maturation does not necessarily signify inferior functional outcomes (eg, maturation failure or abandonment). Rather, reintervention exposes the patient to the risk of additional procedures, as well as potential radiation exposure, interruption of dialysis, hospitalization, and increased TDC dependence, each of which has its own well-documented morbidity as well as increased resource utilization and cost.^26^ It is our impression that the association between high postmaturation reintervention rates and good cumulative functional patency suggests that access abandonment can be avoided or at least delayed in most scenarios with close follow-up and perseverance. Importantly, patients who undergo AVF reintervention subselect for a high-risk population characterized by an increased incidence of subsequent reinterventions frequently requiring serial referral to their access provider. Notwithstanding the intensity of surveillance and provision of care needed to maintain a functional AVF after reintervention, this analysis reaffirms that durable functional outcomes can be achieved with selective remediation.

To further guide management of dysfunctional AVFs after maturation, Table 3 lists success rates for various procedures and how they compare to prematuration outcomes. As expected, procedures classically used to promote maturation (eg, accessory vein ligation, superficialization) were rarely performed after maturation, whereas the relative frequency of reinterventions for fistula stenosis, thrombosis, and central vein stenosis all increased. The proportion of endovascular procedures increased in frequency after maturation but was also associated with an increased success rate (70%).

Analogous to our study, Falk et al^16^ reported that most post-AVF maturation reinterventions were endovascular angioplasty procedures. Improved postmaturation success rates associated with fistula and central vein stenosis indicate that maintaining functional patency is easier than facilitating maturation for venous outflow tract-related pathology. In contrast, procedures to treat AVF thrombosis had poor success rates before (38%) and after (37%) maturation. Although low, these rates are not prohibitive, so thrombectomy procedures can still be considered useful depending on the scenario (eg, limited alternative access options and/or a mature/reliable access). Interestingly, thrombectomy-associated functional success rates do not match the 80% technical success rates reported in the literature.^18^ This highlights that technical success of a procedure does not always accomplish the clinical goal of reintervention. In fact, the pooled technical success rate of various AVF reinterventions has been reported to be as high as 98%, which is superior to the 51% prematuration and 70% postmaturation functional success rates found in this study.^16^^,^^17^

This study has several intrinsic limitations. Our analysis cannot account for center and surgeon selection bias related to initial surgical decisions related to access creation and utilization as well as decisions surrounding when an access was selected for reintervention or abandonment. Moreover, there may be other center and/or surgeon-level effects that confound and bias our results given the study’s nonrandomized sample and lack of standardized clinical decision making, which certainly may have influenced the findings. AVF duplex characteristics are a major variable that influences surgeon management decisions; unfortunately, ultrasound data were unavailable for inclusion in this analysis. We did find that there were center-specific differences in patient characteristics, maturation success rates, cumulative functional patency rates, and rates of some pre- and postmaturation reinterventions. However, there were no center differences in procedure success rates for all types of reinterventions before and after maturation.

We chose to determine clinical success rates for reintervention types rather than per procedure because of the frequency of multiprocedure reinterventions and clinical utility of interventional-based outcomes. However, we concede that this methodology of choosing a primary indication may underestimate frequency and clinical effects of different procedures. Importantly, prosthetic conduits were not included in the HFM study, so our results cannot be extrapolated to AV graft outcomes. Finally, results from this study may not be generalizable or reflect national practice patterns because AVFs were exclusively managed at academic institutions and accessed within highly coordinated dialysis networks, which have been shown to have fewer reinterventions than nonaffiliated or for-profit centers.^21^

In conclusion, reinterventions are common throughout the lifespan of autogenous AV fistula but do not negatively influence the rate of achieving the ultimate goal of successful cannulation and maintenance of a functional access. Maturation occurred in over 70% of all patients undergoing reintervention, and multiple procedures did not appear to reduce the probability of successful cannulation, highlighting the importance of timely remediation. Additionally, reinterventions performed after AVF maturation were reasonably effective at maintaining functional patency. Therefore, because of the high incidence of pre- and postmaturation reintervention, which correspondingly affects maturation time and potential TDC dependence, these factors should be considered and discussed when planning the “right access” for each patient to ensure it aligns with goals of care and patient preferences. More importantly, these results highlight that durable AVF functional outcomes require longitudinal evaluation by an access surgeon, even after successful fistula maturation is achieved.
