# Supplementary material for: The Impact of Reintervention on Arteriovenous Fistula Maturation and Functional Patency in the Hemodialysis Fistula Maturation Study
Source: Kidney Med. 2025 May 26;7(8):101036. doi: 10.1016/j.xkme.2025.101036 (PMC12304899; doi:10.1016/j.xkme.2025.101036)
Supplement: Supplementary File (PDF) — Tables S1-S8. [file mmc1.pdf]

**Table S1. Center-specific patient characteristics and maturation and functional patency outcomes.**

| Variable, No. (%) | Center A *N = 98* | Center B *N = 37* | Center C *N = 53* | Center D *N = 132* | Center E *N = 97* | Center F *N = 86* | Center G *N = 32* | p-value |
|---|---|---|---|---|---|---|---|---|
| **Age** (mean±SD) | 56±12 | 52±12 | 54±15 | 58±15 | 50±13 | 55±13 | 56±11 | <0.001 |
| **Female sex** | 37 (38%) | 11 (30%) | 13 (25%) | 47 (36%) | 25 (26%) | 23 (27%) | 7 (22%) | 0.26 |
| **Race** | | | | | | | | <0.001 |
| White | 33 (34%) | 4 (11%) | 24 (45%) | 65 (49%) | 42 (43%) | 57 (71%) | 21 (66%) | |
| Non-white | 65 (66%) | 33 (89%) | 29 (55%) | 67 (51%) | 55 (57%) | 23 (29%) | 11 (34%) | |
| **BMI** (mean±SD)[a] | 29±8 | 32±8 | 32±8 | 30±7 | 29±7 | 31±7 | 33±9 | 0.02 |
| **Smoking status** | | | | | | | | 0.07 |
| Never | 50 (51%) | 18 (53%) | 25 (48%) | 49 (37%) | 50 (52%) | 45 (52%) | 13 (42%) | |
| Former | 30 (31%) | 9 (27%) | 19 (37%) | 51 (39%) | 31 (32%) | 34 (40%) | 16 (52%) | |
| Current | 18 (18%) | 7 (21%) | 8 (15%) | 32 (24%) | 16 (17%) | 7 (8%) | 2 (7%) | |
| **Diabetes mellitus** | 60 (61%) | 17 (46%) | 26 (49%) | 76 (58%) | 55 (57%) | 53 (62%) | 24 (75%) | 0.19 |
| **Coronary artery disease** | 17 (17%) | 2 (5%) | 19 (36%) | 47 (36%) | 15 (16%) | 31 (36%) | 4 (13%) | <0.001 |
| **Peripheral artery disease** | 8 (8%) | 6 (16%) | 5 (9%) | 25 (19%) | 11 (11%) | 21 (24%) | 6 (19%) | 0.03 |
| **Dyslipidemia** | 59 (60%) | 18 (49%) | 32 (60%) | 77 (58%) | 56 (58%) | 51 (59%) | 6 (19%) | <0.001 |
| **CKD/Pre-emptive access placement**[b] | 31 (32%) | 10 (27%) | 16 (30%) | 43 (33%) | 24 (25%) | 38 (44%) | 20 (63%) | <0.001 |
| **Prior vascular access** | 17 (17%) | 13 (38%) | 10 (19%) | 35 (27%) | 22 (23%) | 14 (16%) | 7 (22%) | 0.13 |
| **Forearm arteriovenous fistula** | 20 (20%) | 14 (38%) | 12 (23%) | 13 (10%) | 31 (32%) | 41 (48%) | 12 (38%) | <0.001 |
| **Successful maturation** | 71 (72%) | 25 (68%) | 36 (68%) | 86 (65%) | 85 (88%) | 71 (83%) | 22 (69%) | 0.002 |
| **Cumulative functional patency**[c] | 48 (49%) | 20 (54%) | 25 (47%) | 65 (49%) | 69 (71%) | 57 (66%) | 17 (53%) | 0.004 |

*[a]BMI, body mass index; [b]CKD, chronic kidney disease (Stage V); [c]Cumulative functional patency outcome determined by fistula use at last known follow up, median follow-up time after maturation 21 [11,31] months.*

**Table S2. Distribution of pre-maturation reinterventions for the entire HFM study cohort and proportion achieving successful maturation.**

| Number of Pre-Maturation Reinterventions | Study Cohort *N = 535 (%)* | AVFs with Successful Maturation *N = 396 (%)* | Mature AVF *%* | p-value |
|---|---|---|---|---|
| 0 | 339 (63%) | 255 (64%) | 75 | 0.50 |
| 1 | 134 (25%) | 95 (24%) | 71 | |
| 2 | 51 (10%) | 39 (10%) | 77 | |
| 3 | 7 (1%) | 3 (<1%) | 43 | |
| 4 | 3 (<1%) | 3 (<1%) | 100 | |
| 5 | 1 (<1%) | 1 (<1%) | 100 | |

**Table S3. Pre-maturation reinterventions performed on arteriovenous fistulas of CKD and ESKD HFM study patients.**

| | CKD Pre-Maturation Interventions (N = 88 interventions in 63 patients) | | ESKD Pre-Maturation Interventions (N = 186 interventions in 133 patients) | |
|---|---|---|---|---|
| **Primary Indication** | **N interventions (% of total)** | **Proportion Successful (%)** | **N interventions (% of total)** | **Proportion Successful (%)** |
| Fistula stenosis | 51 (58%) | 24 (47%) | 115 (62%) | 66 (57%) |
| Thrombosis | 6 (7%) | 3 (50%) | 15 (8%) | 5 (33%) |
| Accessory vein branches | 7 (8%) | 4 (57%) | 12 (7%) | 7 (58%) |
| Central vein stenosis | 5 (6%) | 2 (40%) | 13 (7%) | 7 (54%) |
| Inability to cannulate | 5 (6%) | 4 (80%) | 10 (5%) | 8 (80%) |
| Inflow stenosis | 5 (6%) | 2 (40%) | 8 (4%) | 2 (25%) |
| Hand ischemia | 6 (7%) | 3 (50%) | 10 (5%) | 2 (20%) |
| Other (bleeding, fluid evacuation, infection, aneurysm) | 3 (3%) | 2 (67%) | 3 (2%) | 0 (0%) |

**Table S4. Upper arm and forearm pre-maturation reinterventions performed on arteriovenous fistulas.**

| | Upper Arm Pre-Maturation Interventions (N = 207 interventions in 149 patients) | | Forearm Pre-Maturation Interventions (N = 67 interventions in 47 patients) | |
|---|---|---|---|---|
| **Primary Indication** | **N interventions (% of total)** | **Proportion Successful (%)** | **N interventions (% of total)** | **Proportion Successful (%)** |
| Fistula stenosis | 129 (62%) | 70 (54%) | 37 (55%) | 20 (54%) |
| Thrombosis | 13 (6%) | 5 (39%) | 8 (12%) | 3 (38%) |
| Accessory vein branches | 9 (4%) | 6 (67%) | 10 (15%) | 5 (50%) |
| Central vein stenosis | 16 (8%) | 9 (56%) | 2 (3%) | 0 (0%) |
| Inability to cannulate | 7 (3%) | 5 (71%) | 8 (12%) | 7 (88%) |
| Inflow stenosis | 13 (6%) | 4 (31%) | 0 (0%) | - |
| Hand ischemia | 15 (7%) | 4 (27%) | 1 (2%) | 1 (100%) |
| Other (bleeding, fluid evacuation, infection, aneurysm) | 5 (2%) | 1 (50%) | 1 (2%) | 1 (100%) |

**Table S5. Center-specific pre-maturation reinterventions and success rates.**

| | 274 pre-maturation reinterventions in 198 patients N (%) interventions \| N (%) successful | | | | | | | |
|---|---|---|---|---|---|---|---|---|
| | **Center A** (N=43 int. in 31/98 pat., 32%) | **Center B** (N=19 int. in 12/37 pat., 32%) | **Center C** (N=44 int. in 25/53 pat., 47%) | **Center D** (N=76 int. in 60/132 pat., 45%) | **Center E** (N=33 int. in 26/97 pat., 27%) | **Center F** (N=47 int. in 33/86 pat., 38%) | **Center G** (N=12 int. in 9/32 pat., 28%) | **P \| P** |
| **Fistula stenosis** | 23 (54%) \| 11 (48%) | 12 (63%) \| 5 (42%) | 34 (77%) \| 16 (47%) | 55 (72%) \| 29 (53%) | 16 (49%) \| 12 (75%) | 19 (40%) \| 14 (74%) | 7 (58%) \| 3 (43%) | <0.001 \| 0.24 |
| **Thrombosis** | 3 (7%) \| 1 (33%) | 2 (11%) \| 1 (50%) | 3 (7%) \| 1 (33%) | 2 (3%) \| 1 (50%) | 4 (12%) \| 2 (50%) | 7 (15%) \| 2 (29%) | 0 (0%) | 0.21 \| 0.98 |
| **Accessory vein branches** | 9 (21%) \| 7 (78%) | 1 (5%) \| 0 (0%) | 2 (5%) \| 0 (0%) | 2 (3%) \| 1 (50%) | 1 (3%) \| 1 (100%) | 3 (6%) \| 2 (67%) | 1 (8%) \| 0 (0%) | 0.01 \| 0.18 |
| **Central vein stenosis** | 1 (2%) \| 1 (100%) | 1 (5%) \| 0 (0%) | 1 (2%) \| 1 (100%) | 4 (5%) \| 1 (25%) | 10 (30%) \| 6 (60%) | 0 (0%) | 1 (8%) \| 0 (0%) | <0.001 \| 0.44 |
| **Inability to cannulate** | 0 (0%) | 3 (16%) \| 3 (100%) | 2 (5%) \| 2 (100%) | 2 (3%) \| 0 (0%) | 0 (0%) | 6 (13%) \| 5 (83%) | 2 (17%) \| 2 (100%) | <0.001 \| 0.13 |
| **Inflow stenosis** | 3 (7%) \| 1 (33%) | 0 (0%) | 1 (2%) \| 0 (0%) | 3 (4%) \| 2 (67%) | 1 (3%) \| 0 (0%) | 5 (11%) \| 1 (20%) | 0 (0%) | 0.36 \| 0.87 |
| **Hand ischemia** | 4 (9%) \| 1 (25%) | 0 (0%) | 1 (2%) \| 0 (0%) | 6 (8%) \| 1 (17%) | 1 (3%) \| 1 (100%) | 3 (6%) \| 1 (33%) | 1 (8%) \| 1 (100%) | 0.62 \| 0.48 |
| **Other** (bleed, fluid evac, infection, aneur) | 0 (0%) | 0 (0%) | 0 (0%) | 2 (3%) \| 1 (50%) | 0 (0%) | 4 (9%) \| 1 (25%) | 0 (0%) | 0.06 \| 1.0 |

**Table S6. Distribution of post-maturation reinterventions for the entire study cohort and freedom from abandonment.**

| Number of Post-Maturation Reinterventions | Study Cohort *N = 396 (%)* | Freedom from Access Abandonment[a] *N = 291 (%)* | Patent AVF[a] *%* | p-value |
|---|---|---|---|---|
| 0 | 208 (53%) | 172 (59%) | 83 | 0 vs. 1 or 2: p < 0.001<br><br>3+ vs. 1 or 2: p =0.003 |
| 1 | 75 (19%) | 45 (15%) | 60 | |
| 2 | 42 (11%) | 26 (9%) | 62 | |
| 3 | 27 (7%) | 22 (8%) | 82 | |
| 4 | 15 (4%) | 11 (4%) | 73 | |
| 5 | 15 (4%) | 12 (4%) | 80 | |
| 6 or more | 14 (4%) | 13 (4%) | 93 | |

[a]*At time of last known follow-up*

**Table S7. Demographics and comorbidities of high reintervention patients (5 or more pre- or post-maturation reinterventions).**

| **Variable, No. (%)** | **Less than 5 pre- or post-maturation reinterventions** *N = 352 (89%)* | **5 or more pre- or post-maturation reinterventions** *N = 44 (11%)* | **p-value** |
|---|---|---|---|
| **Age** (mean±SD) | 54±14 | 57±12 | 0.20 |
| **Female sex** | 91 (25.9) | 11 (25.0) | 1.0 |
| **Race** | | | |
| White | 168 (48.6) | 20 (46.5) | |
| Non-white | 178 (51.4) | 23 (53.5) | 0.87 |
| **BMI** (mean±SD)[a] | 29.5±7.2 | 31.1±7.6 | 0.19 |
| **Smoking status** | | | |
| Never | 160 (46.0) | 19 (43.2) | |
| Former | 129 (37.1) | 18 (40.9) | |
| Current | 59 (17.0) | 7 (15.9) | 0.91 |
| **Diabetes mellitus** | 193 (54.8) | 29 (65.9) | 0.20 |
| **Coronary artery disease** | 86 (24.4) | 10 (22.7) | 1.0 |
| **Peripheral artery disease** | 48 (13.6) | 12 (27.3) | 0.03 |
| **Dyslipidemia** | 192 (54.5) | 27 (61.4) | 0.43 |
| **CKD/Pre-emptive access placement**[b] | 118 (33.5) | 9 (20.5) | 0.09 |
| **Prior vascular access** | 81 (23.2) | 15 (34.1) | 0.14 |
| **Forearm arteriovenous fistula** | 100 (28.4) | 3 (6.8) | 0.002 |

*[a]BMI, body mass index; [b]CKD, chronic kidney disease (Stage V)*

**Table S8. Center-specific post-maturation reinterventions and success rates.**

| | **477 post-maturation reinterventions in 188 patients N (%) interventions \| N (%) successful** | | | | | | | |
|---|---|---|---|---|---|---|---|---|
| | **Center A** (N=74 int. in 34/71 pat., 48%) | **Center B** (N=17 int. in 10/25 pat., 40%) | **Center C** (N=81 int. in 26/36 pat., 72%) | **Center D** (N=143 int. in 45/86 pat., 52%) | **Center E** (N=102 int. in 44/85 pat., 52%) | **Center F** (N=37 int. in 20/71 pat., 28%) | **Center G** (N=23 int. in 9/22 pat., 41%) | **P\| P** |
| **Fistula stenosis** | 42 (57%) \| 34 (81%) | 11 (65%) \| 9 (82%) | 59 (73%) \| 49 (20%) | 124 (87%) \| 84 (68%) | 48 (47%) \| 42 (88%) | 23 (62%) \| 18 (78%) | 19 (83%) \| 15 (79%) | <0.001 \| 0.10 |
| **Thrombosis** | 14 (19%) \| 4 (29%) | 4 (24%) \| 1 (25%) | 10 (12%) \| 4 (40%) | 5 (4%) \| 1 (20%) | 20 (20%) \| 10 (50%) | 8 (22%) \| 2 (25%) | 2 (9%) \| 1 (50%) | <0.001 \| 0.75 |
| **Accessory vein branches** | 0 (0%) | 0 (0%) | 0 (0%) | 0 (0%) | 6 (6%) \| 5 (83%) | 1 (3%) \| 1 (100%) | 0 (0%) | <0.001 \| 1.0 |
| **Central vein stenosis** | 9 (12%) \| 8 (89%) | 0 (0%) | 6 (7%) \| 2 (33%) | 10 (7%) \| 8 (80%) | 22 (22%) \| 16 (73%) | 1 (3%) \| 1 (100%) | 1 (4%) \| 1 (100%) | <0.001 \| 0.22 |
| **Inability to cannulate** | 1 (1%) \| 0 (0%) | 0 (0%) | 1 (1%) \| 1 (100%) | 0 (0%) | 0 (0%) | 0 (0%) | 0 (0%) | 0.65 \| 1.0 |
| **Inflow stenosis** | 1 (1%) \| 1 (100%) | 0 (0%) | 0 (0%) | 2 (1%) \| 1 (50%) | 2 (2%) \| 2 (100%) | 3 (8%) \| 2 (67%) | 0 (0%) | 0.07 \| 0.62 |
| **Hand ischemia** | 1 (1%) \| 1 (100%) | 0 (0%) | 1 (1%) \| 0 (0%) | 2 (1%) \| 1 (50%) | 0 (0%) | 1 (3%) \| 1 (100%) | 0 (0%) | 0.84 \| 0.40 |
| **Other** (bleed, fluid evac, infection, aneur) | 6 (8%) \| 2 (33%) | 2 (12%) \| 2 (100%) | 4 (5%) \| 1 (25%) | 0 (0%) | 4 (4%) | 0 (0%) | 1 (4%) \| 1 (100%) | 0.02 \| 0.23 |
